# Does Medicaid Managed Care Help Equalize Racial and Ethnic Disparities in Utilization?

**DOI:** 10.1111/1475-6773.12396

**Published:** 2015-10-12

**Authors:** James Marton, Aaron Yelowitz, Meredith Shores, Jeffery C. Talbert

**Affiliations:** ^1^ Department of Economics and Georgia Health Policy CenterAndrew Young School of Policy StudiesGeorgia State UniversityAtlantaGA; ^2^ Department of EconomicsGatton School of Business and EconomicsUniversity of KentuckyLexingtonKY; ^3^ Department of Pharmacy Practice and ScienceUK College of PharmacyLexingtonKY

**Keywords:** Medicaid, managed care, child health, health care disparities, race/ethnicity, utilization of services

## Abstract

**Objective:**

To estimate the impact of different forms of Medicaid managed care (MMC) delivery on racial and ethnic disparities in utilization.

**Data Source:**

Longitudinal, administrative data on 101,649 children in Kentucky continuously enrolled in Medicaid between January 1997 and June 1999. Outcomes considered are monthly professional, outpatient, and inpatient utilization.

**Study Design:**

We apply an intent‐to‐treat, instrumental variables analysis using the staggered geographic implementation of MMC to create treatment and control groups of children.

**Principal Findings:**

The implementation of MMC reduced monthly professional visits by a smaller degree for non‐whites than whites (3.8 percentage points vs. 6.2 percentage points), thereby helping to equalize the initial racial/ethnic disparity in utilization. The Passport MMC program in the Louisville‐centered region statistically significantly reduced disparities for professional visits (closing the gap by 8.0 percentage points), while the Kentucky Health Select MMC program in the Lexington‐centered region did not. No substantive impact on disparities was found for either outpatient or inpatient utilization in either program.

**Conclusions:**

We find evidence that MMC has the possibility to reduce racial/ethnic disparities in professional utilization. More work is needed to determine which managed care program characteristics drive this result.

There is a vast literature documenting disparities in health care utilization between white and racial/ethnic minorities (Institute of Medicine [IOM] 2002; hereafter “IOM”). Multiple provisions of the Affordable Care Act (ACA) focus on reducing disparities, highlighting its policy relevance. One example is the elevation of the National Center for Minority Health and Health Disparities to full institute status within the National Institutes of Health.^1^ The IOM classifies the causes of these disparities into three categories: differences in insurance coverage, geographic access to providers, and other “system factors” (Balsa, Cao, and McGuire 2007).

One system factor receiving attention is managed care coverage. Managed care could either reduce or exacerbate disparities. Such plans typically coordinate care through closed provider networks and gatekeeper physicians. By reducing choice, such coordination could increase utilization disparities if racial/ethnic minorities face greater transportation and scheduling barriers. There is also concern that gatekeepers may increase disparities if they are less likely to advocate for low‐income, minority patients (Balsa, Cao, and McGuire 2007). Alternatively, if racial/ethnic minorities are more likely to have fragmented care, then an increased emphasis on coordination and uniform practice standards could reduce disparities (Haas et al. 2002; Cook 2007). Coordination may improve because some plans give providers performance‐based bonuses for extending office hours, maintaining an appointment reminder system, and accepting new patients (Marton, Yelowitz, and Talbert 2014).

While there are sizable independent literatures on disparities and MMC, the intersection is small and inconclusive (Cook 2007).^2^ Moreover, little attention is given to inequities in child health (Raphael and Beal 2010), yet there are reasons why a focus on children is warranted: First, children make up nearly half of all Medicaid enrollees, and promoting prevention and instilling healthy behaviors at an early age is economically beneficial.^3^ Second, child ailments differ from adult ailments, and they are worthy of exploration themselves. Children experience few hospitalizations and concentrate their utilization between professional and outpatient services. Finally, since their health needs are fairly basic, minimizing disparities may prove less difficult. The absence of studies focusing on children leaves these issues unaddressed.

Two previous studies focus on the relationship between MMC and disparities among children.^4^ Tai‐Seale, Freund, and LoSasso (2001) used a difference‐in‐differences framework to analyze mandatory MMC enrollment. They found that African American beneficiaries, in comparison to whites, had relatively fewer physician visits after the introduction of MMC. Currie and Fahr (2005) found that an increase in MMC penetration led to a significant decrease in doctor visits among black children and infants with chronic conditions.

Nonrandom selection into health plans is a common challenge associated with identifying the impact of managed care. Often, individuals choose between enrolling in a managed care plan or a traditional fee‐for‐service (FFS) plan. If racial/ethnic minorities select plans differently from whites and this is correlated with subsequent utilization, then changes in disparities cannot be causally attributed to managed care. To address concerns about selection, Tai‐Seale, Freund, and LoSasso (2001) employ a Heckman correction (Heckman 1979). Currie and Fahr (2005) employ multiple instruments for MMC penetration.

## Methods

This paper evaluates the extent to which Medicaid managed care impacts racial/ethnic utilization disparities using a quasi‐experimental approach that exploits the region‐specific implementation of managed care in Kentucky in the late 1990s. As described in detail in Marton, Yelowitz, and Talbert (2014) and Marton and Yelowitz (2015), the Medicaid program in Kentucky was changed from FFS to managed care in two geographically distinct regions surrounding Louisville and Lexington. We compare children initially in each of the two regions before and after this reform with children in the remainder of Kentucky that continued in FFS Medicaid, and we use intention‐to‐treat (ITT) instrumental variables (IV) methods to address nonrandom selection. This comparison allows for some limited degree of speculation regarding the relationship between specific managed care characteristics (i.e., capitated vs. FFS provider reimbursement) and racial/ethnic utilization disparities, but it does not allow us to say anything definitive.

Before laying out a conceptual model describing the impact of MMC on utilization disparities, we describe the implementation of MMC in Kentucky. Starting in November 1997, Kentucky enrolled Medicaid recipients in its two major urban areas (Louisville and Lexington) and surrounding counties in separate managed care organizations (MCOs). The MCO covering the Louisville region consisting of 16 counties was named the *Passport Health Plan* (Passport) and was designed around the University of Louisville hospital network. The MCO covering the Lexington region consisting of 21 counties was named the *Kentucky Health Select Plan* (KHS) and was designed around the University of Kentucky hospital network. Medicaid recipients within each region were mandated to join their region's respective MCO.^5^

KHS dissolved in June 2000 and its enrollees returned to FFS Medicaid coverage, while the Passport MCO continued operations. Medicaid recipients outside of Passport's region remained in FFS Medicaid until late 2011, when Kentucky introduced statewide MMC.^6^ Our timeframe spans January 1997 through June 1999, which implies that we have 10 months of prereform and 20 months of postreform data (during which time both MCOs were operating).

Both MCOs were responsible for covering their Medicaid enrollees in exchange for state capitation payments. Passport reimbursed primary care providers (PCPs) via capitation, with the rate adjusted for patient case mix. Thus, the marginal revenue from an additional visit was zero. Hospitals were reimbursed on a per diem basis using the Medicaid fee schedule with a 10 percent withhold. In contrast, KHS reimbursed both physicians and hospitals on an FFS basis using the Medicaid fee schedule with a 20 percent withhold. This implies positive marginal revenue for additional visits. Another difference between the MCOs was the way in which they performed basic administrative functions, such as claims processing and case management. Passport outsourced these responsibilities to an existing administrative service organization. KHS handled these responsibilities internally, despite inexperience at managing such a network.

How might these MCOs impact utilization disparities conceptually? Managed care plans coordinate care through the use of closed provider networks, gatekeeper physicians, and provider financial incentives. Financial incentives such as capitated (prospective) payment for services typically lead to reductions in enrollee utilization. If racial/ethnic minorities face greater transportation barriers, then such care coordination could lead to larger reductions in utilization and result in an increase in disparities. For example, suppose care coordination is implemented by requiring each enrollee to see his or her gatekeeper physician (rather than any PCP) before receiving specialist care. If an enrollee only has access to transportation on certain days or at certain times, then he or she may have more difficulty seeing a specific gatekeeper to get a referral than he or she would have in seeing any PCP for a referral or being able to schedule an appointment with the specialist directly. Thus, such enrollees may incur a larger reduction in utilization than those without transportation issues.

On the other hand, if minorities are more likely to have fragmented care, then the care coordination provided by an MCO may lead to smaller net reductions in utilization for minorities as compared to whites (Cook 2007). For example, suppose that with Medicaid FFS coverage, minorities are more likely to wait until medical problems advance to the point of requiring immediate emergency room (ER) care. If the tools of managed care increase the amount of primary care these minorities receive, then their utilization of ER and inpatient care may fall. Thus, minorities with fragmented care would have reductions in some utilization categories (ER, inpatient) that are offset to some degree by increases in another utilization category (primary care visits). We might expect a white enrollee without fragmented care to experience reductions in ER and inpatient care as a result of MMC, but a smaller (or no) increase in primary care. A comparison of these two types of enrollees would suggest a reduction in their initial utilization disparity.

It is possible that reductions in utilization may not come from efficiency gains, but instead from reductions in access that could negatively impact health. Thus, MMC may reduce access in such a way that disparities are reduced (by reducing white enrollee utilization by a greater degree than non‐white utilization), but this reduction in access could lead to reductions in health status for all enrollees because everyone is receiving less care.

Our analysis uses de‐identified Medicaid claims and enrollment data from the Kentucky Cabinet for Health and Family Services.^7^ We received IRB approval from the University of Kentucky and the Kentucky Cabinet for Health and Family Services. We extract the 101,649 children (aged 0–19 years) continuously enrolled in Medicaid for all 30 months between January 1997 and June 1999. Because MMC was introduced in November 1997, we have 20 months of postreform data. Analyzing a longer postreform period was not possible due to a change in the vendor managing the Kentucky Medicaid information systems.^8^

In terms of measuring race, Medicaid enrollees were asked to self‐report their race by checking one of a number of options: white, black, Asian, Hispanic, Indian, or other. Some did not answer the question, so their response was coded as “no answer” in the Medicaid database. We aggregate all options other than white into a single category called “non‐white.” In practice, the number of children enrolled in Kentucky Medicaid that do not self‐report as being “white” or “black” is very small. Out of 101,649 respondents, 94.9 percent self‐reported themselves as white or black. Of the remaining 5.1 percent (5,188 children), a majority (3,682) did not provide a response. Roughly 1.5 percent of children self‐report as a race other than white or black.

We focus on three broad measures of utilization: inpatient, outpatient, and professional services. Inpatient services are defined to be services delivered in a hospital with an overnight stay, while outpatient services are services delivered in clinics or hospitals in which there is no overnight stay (such as an ER visit). Professional services typically represent physician services, but they could also include services provided at locations other than physician offices, such as dental or public health clinics. We focus on these outcome measures for two reasons: our desire to be consistent with previous work evaluating the implementation of MMC in Kentucky and the fact that these categories of utilization were reimbursed differently by the two MCOs.

The Kentucky MMC transition essentially creates a quasi‐experiment, where individuals in the Passport and KHS regions were largely compelled to participate in MMC, and individuals in the remainder of Kentucky did not have the option of participating. A straightforward empirical model that estimates the effect of MMC would be:

(1) $${\text{UTIL}}_{it}=\beta_{0}+\beta_{1}{\text{MMC}}_{it}+\gamma X_{it}+\alpha_{i}+\delta_{t}+\varepsilon_{it}$$

where the subscript *i* represents individuals and *t* represents time period. The outcome variable UTIL_*it*_ is a monthly measure of professional, outpatient, or inpatient utilization, MMC_*it*_ is an indicator for monthly participation in MMC, and *X*_*it*_ indicate individual characteristics that vary over time (such as age or family structure). The coefficient *α*_*i*_ represents individual fixed‐effects (and nets out any time‐invariant individual characteristic, such as underlying health) and *δ*_*t*_ is a time fixed‐effect for each of the 30 months between January 1997 and June 1999.^9^ With these controls, an estimate of *β*_1_ < 0 would suggest that MMC reduces utilization.

Similarly, one could estimate the separate impacts of Passport and KHS with a variant of equation (1):

(1') $${\text{UTIL}}_{it}=\beta_{0}+\beta_{1}{\text{PASSPORT}}_{it}+\beta_{2}{\text{KHS}}_{it}+\gamma X_{it}+\alpha_{i}+\delta_{t}+\varepsilon_{it}$$

where PASSPORT_*it*_ and KHS_*it*_ are indicators for monthly participation in a particular MMC program (and a child can only participate in—at most—one MMC program in a given month). Much like specification equation (1), one expects participation in either Passport or KHS to reduce utilization, and a finding of *β*_1_ ≠ *β*_2_ would indicate a differential impact associated with each plan.

A drawback of either specification relates to selection bias. First, children in Kentucky often move from one county to another. An inference problem emerges if families systematically move in response to the enactment of MMC. For example, if a family with high expected utilization moves away from a county with MMC to one with FFS, then the coefficient estimates will be biased upward.^10^ In other words, if families with sick children are unsatisfied with MMC and move away, then our model would predict large reductions in utilization because of MMC, when in fact that result is driven by changes in the composition of the health status patients the managed care plans are responsible for. Second, although participation in MMC was intended to be compulsory, in practice, it was not. If MMC participants are selected nonrandomly, the resulting estimates will be biased.

An approach to overcome such selection bias is to use an ITT IV approach, with the managed care status based on a child's initial county of residence (in month 1) and actual time period as the instrument, as in Bindman et al. (2005). If a child was initially located in the Passport or KHS region in January 1997, then the instrument—denoted below as MMC_ELIG_*it*_—would equal to one for the period November 1997 to June 1999, and zero otherwise. For children initially outside of these regions, the instrument would always be equal to zero. Analogous to specification equation (1), the first‐stage regression would predict MMC participation:

(2) $$\text{Public}\;{\text{Coverage}}_{ict}=\beta_{0}+\beta_{LIHP}\text{Post}2011_{t}^{*}\text{Expansion}\;{\text{County}}_{c}+\beta_{1}X_{ict}+\mu {\text{Year}}_{t}+\Omega {\text{County}}_{c}+\varepsilon_{ict}$$

This instrument reflects the ITT framework in econometrics and clinical trials with incomplete compliance (Angrist and Pischke 2008).^11^ In essence, predicted values for MMC participation rather than actual values are used to estimate the effect on utilization in a specification similar to equation (1). Similarly, we construct instruments PASSPORT_ELIG_*it*_ and KHS_ELIG_*it*_ based on the whether the child was initially in a Passport or KHS region, before or after November 1997. We estimate such models with the *xtivreg* commands in Stata 13.0 (StataCorp 2013).

It is straightforward to extend the IV specifications to explore disparities. Here, we reiterate that the IOM defines disparities as racial or ethnic differences in the quality of health care that “are not due to access‐related factors or clinical needs, preferences, and appropriateness of intervention.” In our study, access‐related factors are held constant because all children are continuously enrolled. Furthermore, the IOM defines patient preferences as “patients’ choices regarding health care that are based on a full and accurate understanding of treatment options.” We control for household preferences, understanding of treatment options, and health care needs through the inclusion of individual child fixed‐effects. Although each of these factors varies from one child to the next, they are likely fixed over our 30 months timeframe. Despite the fact that a child's race/ethnicity is time invariant, and subsumed in the individual fixed effect (*α*_*i*_), the interaction of race/ethnicity and MMC status does change and can be included in the specification. Thus, equation (1) can be modified to explore racial disparities as follows:

(3) $${\text{UTIL}}_{it}=\beta_{0}+\beta_{1}{\text{MMC}}_{it}+\beta_{2}{\text{MMC}}_{it}\cdot {\text{NONWHITE}}_{i}+\gamma X_{it}+\alpha_{i}+\delta_{t}+\varepsilon_{it}$$

where the coefficient *β*_2_ represents the interaction of MMC participation and non‐white status and represents the additional impact—positive or negative—of MMC utilization on non‐whites relative to whites. A positive coefficient would indicate an equalizing effect of MMC, while a negative coefficient would indicate that disparities get larger. Each specification is estimated as a linear probability model; interaction terms in logit or probit models are often misinterpreted (Ai and Norton 2003). Instruments can be formed as in the previous specification, where MMC_ELIG_*it*_ · NONWHITE_*it*_ would be an additional IV. Finally, how disparities interact with different forms of MMC is modeled in (3’):

(3’) $${\text{UTIL}}_{it}=\beta_{0}+\beta_{1}{\text{MMC}}_{it}+\beta_{2}{\text{MMC}}_{it}\cdot {\text{NONWHITE}}_{i}+\gamma X_{it}+\alpha_{i}+\delta_{t}+\varepsilon_{it}$$

where the coefficients *β*_2_ and *β*_4_ represent the racial disparities associated with the different plans.

## Results

Table 1 provides descriptive statistics by region and race. In the full sample, described in the far left panel, 20.5 percent initially lived in the Passport region, 12.3 percent in the KHS region, and the remaining 67.2 percent in the rest of Kentucky. There are clear regional differences; for example, the percentage of children that experienced a move between counties at any time over the 30 months period is lower in the Passport region (13.1 percent) than the KHS region (25.9 percent), although many of these county‐to‐county moves are within managed care regions. Passport also contains Kentucky's largest urban area—Louisville—which has greater racial diversity than the rest of the state. The differences between Passport and either KHS or the rest of Kentucky reported in the far left panel are all statistically significant at the 1 percent level except for differences in gender. In addition to regional differences, there are differences by race. Roughly 21.5 percent of the sample is non‐white. Among racial/ethnic minorities, the middle panel illustrates that the probability of a move across counties is lower (even within managed care region) and children are significantly younger.

**Table 1:** Summary Statistics

	*Full Sample*				*Non‐White Only*				*White Only*			
	*All Regions*	*Initially Passport Region*	*Initially KHS Region*	*Initially Other Region*	*All Regions*	*Initially Passport Region*	*Initially KHS Region*	*Initially Other Region*	*All Regions*	*Initially Passport Region*	*Initially KHS Region*	*Initially Other Region*
No. of children	101,649	20,836	12,509	68,304	21,829	10,815	2,964	8,050	79,820	10,021	9,545	60,254
% any county move	18.8	13.1	25.7	19.2	12.2	5.9	18.1	18.5	20.6	20.9	28.1	19.3
Age (Jan. 1997)	6.9	6.8	7.0	6.9	6.2	6.5	6.1	5.8	7.1	7.0	7.2	7.0
% non‐white	21.5	51.9	23.7	11.8	100	100	100	100	0	0	0	0
% female	47.6	47.8	47.0	47.6	46.2	47.9	45.9	4.4	47.9	47.7	47.4	48.0
Number of siblings	0.83	0.94	0.77	0.81	0.85	1.02	0.84	0.61	0.83	0.85	0.75	0.83
MMC enrollment (Jan. 1999)	29.4%	89.0	86.4	0.8	57.2	91.0	86.5	1.1	21.8	86.8	86.3	0.8
Professional (pre)	33.7%	28.6	31.8	35.6	28.7	23.1	28.2	36.3	35.1	34.6	32.9	35.5
Professional (post)	31.3%	21.0	30.7	34.6	25.6	18.2	26.5	35.3	32.9	24.0	32.0	34.5
Outpatient (pre)	9.7%	7.5	9.3	10.4	8.7	6.9	8.9	10.9	10.0	8.1	9.4	10.3
Outpatient (post)	8.2%	3.9	7.6	9.6	6.3	3.3	7.3	9.8	8.7	4.4	7.7	9.6
Inpatient (pre)	0.7%	0.5	0.4	0.7	0.7	0.5	0.6	1.0	0.6	0.5	0.4	0.7
Inpatient (post)	0.4%	0.3	0.3	0.5	0.4	0.3	0.4	0.6	0.4	0.3	0.3	0.5

*Notes*.

The prereform time period is January 1997 to October 1997, while the postreform time period is November 1997 to June 1999. *Source*: De‐identified, linked Medicaid claims and enrollment data provided by the Kentucky Cabinet for Health and Family Services.

A comparison of the middle panel with the far right panel shows that there are racial/ethnic disparities in utilization prior to the transition to MMC. While 10 percent of whites had outpatient utilization prior to the transition, just 8.7 percent of non‐whites did. Additionally, 35 percent of whites had a professional visit, compared with just 29 percent of non‐whites. Within Passport, the prereform disparities are evident, especially for professional visits: there is a 12 percentage point gap, as well as a gap for outpatient visits (but not inpatient). Prereform gaps exist in the KHS region as well, but they are not as large.

**Figure 1:**
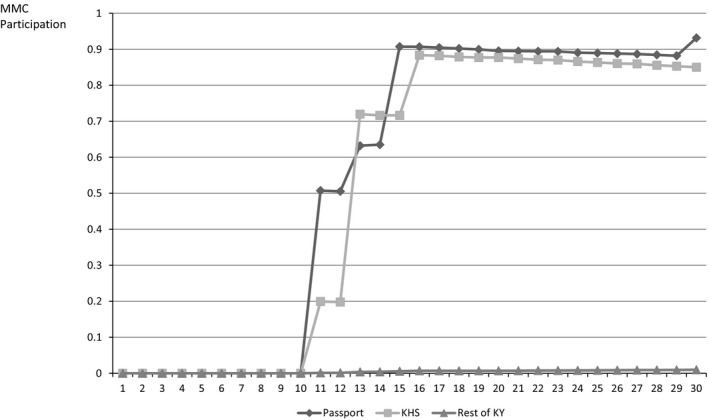
MMC Enrollment Stratified by Initial County of Residence *Notes*: MMC participation rates computed from 101,649 continuously enrolled children over 30 months (i.e., 3,049,470 child‐months). Passport, KHS, and Rest of KY are initial county of residence in month 1 (i.e., January 1997). Transition to Medicaid managed care occurred between November 1997 and April 1998 in the Passport and KHS regions (i.e., months 11–16). *Source*: De‐identified, linked Medicaid claims and enrollment data provided by the Kentucky Cabinet for Health and Family Services.

Turning to the implementation of managed care, Figure 1 illustrates the evolution of MMC enrollment of children over time, stratified by initial county of residence (initially in a Passport county, initially in a KHS county, or initially in another county). Following the introduction of MMC, enrollment grew rapidly over the next 6 months, with roughly 90 percent of children initially in MMC counties enrolling in MMC. Virtually no children initially residing outside of these regions were enrolled in MMC, however. Taken together, these results suggest that compliance was very high but not complete.^12^

Returning to Table 1, we now focus on comparisons before and after the transition to managed care. One can observe reductions in monthly professional, outpatient, and inpatient utilization. These utilization declines differ in magnitude for Passport, KHS, and the rest of Kentucky, thus foreshadowing our principal finding: the transition to MMC reduces, but does not completely eliminate, initial disparities. Within Passport, professional utilization falls by nearly 11 percentage points for whites and 5 percentage points for non‐whites. Thus, the shift from FFS to MMC through Passport provides an equalizing effect on the initial racial utilization disparities. Within the KHS region, however, the declines in utilization are nearly identical by race, which suggests a heterogeneous impact of MMC.

Given concerns related to selection bias caused by family county of residence and/or participation in MMC being based on child health status, our regression results presented below are based on IV in all cases. Before turning to disparities, Table 2 shows the effects of MMC on utilization. The first set of columns compares children in any form of managed care to those in FFS. We find that participation in MMC statistically significantly reduces the probability of having any monthly professional or outpatient utilization. Relative to the initial baseline monthly utilization rate of 33.7 percent for professional visits, MMC reduces utilization by 5.2 percentage points, a statistically significant reduction of more than 15 percent (= [.052/.337]*100). The probability of any monthly outpatient utilization falls by 2.7 percentage points, a statistically significant reduction of more than 27 percent. The probability of any monthly inpatient utilization increases, but the magnitude—0.04 percentage points—implies that fewer than 50 out of the more than 100,000 children experience hospitalizations due to MMC in a given month. The second set of columns distinguishes Passport from KHS. The probability of any monthly professional utilization fell by nearly 8.0 percentage points in Passport, a statistically significant decline of 28 percent. In contrast, professional utilization fell insignificantly by 0.02 percentage points in KHS. The decline in the probability of any monthly outpatient utilization was also larger in Passport: 3.5 percentage points (or 46 percent) in Passport versus 1.2 percentage points (or 13 percent) in KHS.

**Table 2:** How Does MMC Affect Utilization?

	Professional Utilization	Outpatient Utilization	Inpatient Utilization	Professional Utilization	Outpatient Utilization	Inpatient Utilization
MMC	−5.15*** (0.13)	−2.67*** (0.08)	0.04* (0.02)			
Passport				−7.99*** (0.15)	−3.48*** (0.10)	−0.02 (0.03)
KHS				−0.02 (0.20)	−1.20*** (0.13)	0.15*** (0.03)

*Notes*.

Unit of observation is 3,049,470 child‐months. Sample consists of 101,649 children in Kentucky's 120 counties who were continuously enrolled in Medicaid for the 30 months from January 1997–June 1999. All specifications were estimated using instrumental variables and additionally include child fixed‐effects, as well as controls for child's age, family structure, and time fixed‐effects. Standard errors in parentheses. The magnitude of the effect is illustrated by the first column: enrollment in MMC reduces the likelihood of monthly professional utilization by 5.15 percentage points. The interpretation of the other coefficients is similar.

***p < 1%, **p < 5%, *p < 10%. *Source*: De‐identified, linked Medicaid claims and enrollment data provided by the Kentucky Cabinet for Health and Family Services.

We next turn to racial/ethnic disparities in Table 3. Evidence of such disparities is found by interacting the MMC participation indicator with an indicator for the child being non‐white. As in Table 2, we examine MMC as a whole and also split out Passport and KHS. The results suggest that while reducing overall utilization, MMC provides an equalizing effect by race/ethnicity. This effect is statistically significant for Passport and larger than the effect estimated for KHS. For example, the probability of any monthly professional utilization drops by 12.4 percentage points for whites when Passport is implemented, but only 4.4 percentage points (= −0.1241 + 0.0801) for non‐whites. These drops correspond to a 36 percent reduction in professional utilization for whites and a 19 percent reduction for non‐whites (who start at a lower baseline utilization rate). The professional utilization result for KHS—while opposite signed and suggesting an increase in racial disparities—is smaller than the estimated effect for Passport. This table also suggests that Passport does not equalize outpatient disparities: the probability of any monthly outpatient utilization falls by 3.8 percentage points for whites, and 3.2 percentage points for non‐whites, both reductions of approximately 47 percent. Finally, the results for inpatient stays are statistically insignificant.

**Table 3:** How Does MMC Impact Racial/Ethnic Disparities?

	Professional Utilization	Outpatient Utilization	Inpatient Utilization	Professional Utilization	Outpatient Utilization	Inpatient Utilization
MMC	−6.23*** (0.17)	−2.61*** (0.11)	0.08*** (0.03)			
MMC × Nonwhite	2.44*** (0.31)	0.06 (0.20)	0.02 (0.05)			
Passport				−12.41*** (0.22)	−3.81*** (0.14)	0.02 (0.04)
Passport × Nonwhite				8.01*** (0.27)	0.59*** (0.17)	−0.07 (0.05)
KHS				0.25 (0.22)	−1.27*** (0.14)	0.17*** (0.04)
KHS × Nonwhite				−1.16*** (0.44)	0.27 (0.28)	−0.11 (0.07)

*Notes*.

Unit of observation is 3,049,470 child‐months. Sample consists of 101,649 children in Kentucky's 120 counties who were continuously enrolled in Medicaid for the 30 months from January 1997–June 1999. All specifications were estimated using instrumental variables and additionally include child fixed‐effects, as well as controls for child's age, family structure, and time fixed‐effects. Standard errors in parentheses. The magnitude of the results is illustrated by the first column: enrollment in MMC reduces the likelihood of monthly professional utilization by 6.23 percentage points for white children, and by 3.79 percentage points for non‐white children (i.e., −6.23 + 2.44). The interpretation of the other coefficients is similar.

***p < 1%, **p < 5%, *p < 10%. *Source*: De‐identified, linked Medicaid claims and enrollment data provided by the Kentucky Cabinet for Health and Family Services.

## Discussion

Our key finding suggests that the introduction of the Passport plan was associated with an equalizing effect across race/ethnicity for professional utilization. The reduction in disparities we observe is caused by a smaller reduction in professional utilization for minorities as compared to whites within Passport. Because our professional utilization outcome includes primary care, this result is consistent with improvements in care coordination due to managed care leading to more regular primary care utilization among minorities (Haas et al. 2002; Cook 2007).^13^ This in turn offsets for minorities more so than whites the general reduction in utilization typically produced by MMC and results in fewer disparities. Such a mechanism is consistent with one of the disparity reduction channels described in our conceptual model.

The IV model is a transparent specification that controls for selection bias related to migration and plan choice. We also estimated alternative specifications without IVs that do not account for selection bias; our conclusions remain essentially unchanged. For example, when examining professional utilization, we find reductions of 11.5 percentage points in the probability of any monthly utilization for white children from Passport, and 4.1 percentage points for non‐white children. If instead we use a fixed‐effects logistic regression (using the *xtlogit* command in Stata 13.0), we arrive at similar conclusions. Finally, we estimated the reduced‐form model (by regressing utilization on the instrument) and find slightly different magnitudes: Passport reduces the probability of any monthly professional utilization by 9.6 percentage points for whites and 3.9 percentage points for non‐whites. The robust findings across specifications confirm the intuition provided in the summary statistics that Passport reduced disparities.

One limitation of our study is the difficulty of separating out racial and ethnic minorities. Of the 101,649 children in our sample, 79,820 (78.5 percent) are white, 16,641 (16.4 percent) are African American, 3,682 are unknown (3.6 percent), and the remaining 1,506 (1.5 percent) are Hispanic, Other Race, Asian, or Indian. We estimated models restricting the sample to the 96,461 white and African American children; the results are virtually identical.

Another common administrative data limitation is that we have relatively few child or household explanatory variables. By including child fixed‐effects, however, our estimate of the effect of MMC controls for any time‐invariant characteristic—such as the child's latent health or the family's economic circumstances. Even if time‐varying factors—such as changes in health—do affect utilization, such factors are unlikely correlated with the staggered implementation of MMC and would therefore not affect our inferences on the impact of MMC on disparities. Nonetheless, such factors are of independent interest, and we cannot analyze them here. Additionally, our administrative data come from quasi‐experiment that occurred almost 20 years ago, so it is somewhat dated. The Passport program continues to exist in much the same form and was expanded statewide with the rollout of Kentucky's ACA Medicaid expansion in 2014.

As previously mentioned, a comparison of the Passport and KHS programs allows for some limited degree of speculation regarding the relationship between specific managed care characteristics and racial/ethnic utilization disparities. The primary differences between the Passport and KHS plans in terms of plan characteristics included differences between their choice of PCP reimbursement mechanism and how they handled basic administrative functions. Although the Passport plan equalized utilization to a greater degree than the KHS plan, we cannot say for sure if this result is driven by differences in one or both of these two plan characteristics.^14^ In addition, we cannot rule out that there is some other unobserved difference between the two plans besides these two plan characteristics that is driving this result.

A final limitation is that we use retrospective/observational data. Although the children were not participants in a randomized controlled trial, the fact that MMC was implemented on a staggered schedule creates a plausible quasi‐experiment. Our core results are similar to the ones generated without controlling for selection bias, suggesting that there is little cause from concern about nonrandom enrollment of MMC in Kentucky.

When racial utilization disparities are reduced due to a smaller reduction in care for non‐whites as opposed to whites, a natural concern is that these utilization reductions are being driven by reductions in access to needed care, rather than more efficient delivery of care. If the reductions are being driven by access to needed care, then policy makers face a trade‐off between the positive features associated with MMC (reductions in spending, reductions in disparities) and this potential negative impact on enrollee health status. Marton, Yelowitz, and Talbert (2014) investigate this access issue in the context of Kentucky's implementation of MMC and find that the number of unique providers in the Medicaid claims data does not change after the introduction of MMC. This suggests that the observed reduction in disparities may not be driven by limited access, though this finding is certainly not definitive and is worthy of future independent investigation.

## Supporting information

Appendix SA1: Author Matrix.

## Notes

1 Robert Wood Johnson Foundation (2011) provides further discussion of provisions related to disparities in the ACA.

2 For further discussion on disparities, see the special June 2012 issue of *Health Services Research*, “Measuring and Analyzing Health Care Disparities,” as well as Cook et al. (2010). For discussion of MMC, see Sparer (2012), Duggan and Hayford (2013), Harman et al. (2011, 2014), Bindman et al. (2005), and Hu, Chou, and Deily (2015).

3 Childhood Medicaid coverage increases tax revenue and health and reduces hospitalizations in adulthood (Brown, Kowalski, and Lurie 2014; Wherry et al. 2015).

4 For studies focusing on adults, see Haas et al. (2002), Balsa, Cao, and McGuire (2007), Cook (2007), and Alegría et al. (2012).

5 This discussion draws on Bartosch and Haber (2004).

6 For more about the statewide expansion, see http://medicaidmc.ky.gov/Pages/index.aspx (accessed 10/16/2014)

7 Kentucky Medicaid and CHIP administrative data have been used in previous studies, including Kenney et al. (2007, 2011).

8 An important caveat with encounter/claims data is that providers may under‐report service use with capitation contracts (Tai‐Seale, Freund, and LoSasso 2001), which in turn could bias measurement of service use in MMC versus FFS. Marton, Yelowitz, and Talbert (2014) note that PCPs in Passport were given an encounter claims bonus of roughly $1 for every non‐FFS encounter submitted, which reduces this incentive. Moreover, there is no reason to believe these filing incentives should vary by race/ethnicity.

9 Some earlier evaluations, such as Tai‐Seale, Freund, and LoSasso (2001), use separate samples before and after the implementation of MMC. Thus, they cannot include individual fixed‐effects because they cannot follow people over time.

10 This “migration endogeneity” is an issue recognized in Aizer, Currie, and Moretti (2007). They use similar methods to control for endogeneity of location. Gelbach (2004) and Schwartz and Sommers (2014) investigate state‐to‐state migration with respect to safety net programs.

11 Regressing utilization directly on the eligibility instrument is the reduced‐form estimate, and akin to the “difference‐in‐differences” estimator (Yelowitz 1995).

12 A few categories of Medicaid recipients were explicitly carved out of managed care coverage, including those in nursing or psychiatric facilities for an extended stay, those served under home/community‐based waivers, and those who must “spend down” to meet Medicaid eligibility income criteria. In practice, few children fall into these categories.

13 Besides a relatively larger increase in primary care utilization, another reason why managed care may possibly reduce aggregate utilization less for racial/ethnic minorities is the potential for minorities to benefit to a relatively larger degree from Medicaid standards regarding adequate MCO geographic provider networks (Cook 2007).

14 One could more directly test for plan differences if we had four otherwise identical plans, where plan A deviated by capitated PCPs only, plan B deviated by outsourcing administrative functions only, plan C deviated in both ways, and plan D did neither. Our study is essentially a comparison of plan C and plan D.
