# Supplementary material for: Does Medicaid Managed Care Help Equalize Racial and Ethnic Disparities in Utilization?
Source: Health Serv Res. 2015 Oct 12;51(3):872–91. doi: 10.1111/1475-6773.12396 (PMC4874821; doi:10.1111/1475-6773.12396)
Supplement: Supplementary file 1 — Appendix SA1: Author Matrix. [file HESR-51-0872-s001.pdf]

HSR

# REQUIRED INFORMATION AND FORM FOR ACCEPTED MANUSCRIPTS AT HSR [SEPT., 2009]

## *HSR Authorship Responsibility, Disclosures, and Acknowledgments*

**General instructions:**
The corresponding author is responsible for coordinating with each author to obtain all information disclosed on this form. This information is required to be submitted in the section on Manuscript Central labeled for the editors only, i.e., as supplementary files not for review, at the time of the first revision and should be updated as necessary for subsequent revisions or in response to queries from the editor-in-chief.

**Each author** must read and provide the information to the corresponding author on:
(1) Authorship Responsibility, Criteria, and Contributions,
(2) Financial Disclosures, and
(3) Other Disclosures,

The **corresponding author** must complete and submit on behalf of all authors the summary form, affirming the information provided is accurate. The submitted form should contain the answers for the corresponding author on items 1-3 and summarize all authors' answers in the (4) Author and Other Contributors Section and (5) Acknowledgment/Disclosure Statement.

This form is available online at HSR.org *and* http://mc.manuscriptcentral.com/hsr. If necessary, it may be photocopied and distributed to coauthors or other contributors.

**NOTE:** We require this form to be completed before publication. The joint acknowledgement/disclosure statement and author matrix will be published electronically; the joint acknowledgement/disclosure statement [including any contributors to be acknowledged] will also appear in the print version. Each form will be approved by the editors to make sure that all necessary and relevant information that readers need to know to evaluate the work published in HSR has been reflected in the acknowledgement/disclosure paragraph; detailed disclosures will not be published.

The corresponding author should collect the disclosure information from each co-author and provide that information in the appropriate box below. The corresponding author should then prepare a draft joint acknowledgement/disclosure statement along with the author matrix. Each author should review and approve of the final joint statement and authorship information.

The wording of the acknowledgement/disclosure statement and author matrix may undergo some negotiation with the editors, either to eliminate some material that is not needed or to include some that should be disclosed. As only the corresponding author can upload information to Manuscript Central, he or she will coordinate the process. All authors should submit drafts of their disclosures electronically to the corresponding author. The corresponding author may submit them and a draft of the joint statement electronically at any time during our processing of the manuscript. This material will be reviewed for consistency and completeness when the manuscript is close to acceptance.

**Health Services Research**
**Certification and Disclosures by Authors**

**Manuscript Number: HSR-** 14-0680
[After submitting your manuscript online, your manuscript is assigned a number that must be entered in the space above].

Corresponding Author: James Marton

**Please fill in the following table: AUTHOR ORDER AND CONTACT INFORMATION**

| Authors, in preferred order for publication | Telephone Number | Fax Number | e-mail address |
|---|---|---|---|
| James Marton | 404-413-0256 | 404-413-0145 | marton@gsu.edu |
| Aaron Yelowitz | 859-257-7634 | (859) 323-1920 | aaron@uky.edu |
| Meredith Shores | 859-257-7634 | (859) 323-1920 | meredith.shores@uky.edu |
| Jeffery C. Talbert | 859-323-7141 | 859-323-0803 | jtalb1@email.uky.edu |
| | | | |
| | | | |
| | | | |
| | | | |
| | | | |
| | | | |
| | | | |
| | | | |
| | | | |
| | | | |
| | | | |

**INSTRUCTIONS: EACH AUTHOR MUST READ SECTIONS 1-3 AND PROVIDE FULL DISCLOSURE TO THE CORRESPONDING AUTHOR. THE SUBMITTED FORM SHOULD PROVIDE INDIVIDUAL INFORMATION FOR THE CORRESPONDING AUTHOR AND SUMMARIZE ALL AUTHORS' INFORMATION.**

**1. Authorship Responsibility, Criteria, and Contributions.** Each author should meet all criteria below (A, B, C, and D) and should affirm his or her general and specific contributions to the corresponding author. The corresponding author should then complete the information for him/herself by checking the appropriate boxes below and summarize the information for all authors in section 4 and 5. This summary statement should be consistent with and reflect all significant disclosures of each of the individual authors. Research and manuscripts may depend critically on various individuals who do not meet all the criteria for authorship; they should be acknowledged as contributors in section 4 below.

☑ A. I certify that

- the manuscript represents valid work and that neither this manuscript nor one with substantially similar content in which I am an author has been published or is being considered for publication elsewhere, except as described in an attachment, and that copies of closely related manuscripts have been provided to the editors or HSR; and
- [for papers with more than 1 author], I agree to allow the corresponding author to serve as the primary correspondent with the editorial office, to review the edited typescript and proof, and to make decisions regarding release of information in the manuscript to the media, federal agencies, or both; or,
- [for papers for which I am the only author] I will be the corresponding author and agree to serve in the roles described above.

☑ B. I have given final approval of the submitted manuscript and each of its revisions, if any.

☑ C. I have participated sufficiently in the work to take public responsibility for (check 1 or 2 below)

☑ 1. the whole content.

☐ 2. part of the content: (fill in as appropriate)

limited to

all but

☑ D. To qualify for authorship, you must check at least 1 box for *each* of the 2 categories of contributions listed below. For examples of contributions that qualify for acknowledgment but not for authorship, see section 4 below.

**I have made substantial contributions to the intellectual content of the paper as described below**.

1. (check at least 1 of the 3 below)

☑ a. conception and design
☑ b. acquisition of data
☑ c. analysis and interpretation of data

2. (check at least 1 of 2 below)

☑ a. drafting of the manuscript
☑ b. critical revision of the manuscript for important intellectual content

**2. Financial Disclosure.** For the purposes of this disclosure and the one below, "conflicts of interest" include not only situations in which decisions and judgment have been influenced, but also those that may, if subsequently disclosed or uncovered, lead readers to question whether decisions or judgment may have been influenced by such situations. We anticipate that the vast majority of "conflicts," if disclosed in advance, will have little bearing on a reader's assessment of the research and no bearing on our decision to publish. Except in cases in which all the authors certify they have nothing to disclose, the corresponding author will submit a brief acknowledgement/disclosure of the conflicts of all the authors in Section 4.

**Please check the appropriate boxes below (applies to the past five years and foreseeable future):**

☐ a. I have no relevant financial interests pertaining to this manuscript.

☑ b. I certify that all my conflicts of interest, including specific financial interests and relationships and affiliations relevant to the subject matter or materials discussed in the manuscript (e.g., employment / affiliation, grants or funding, consultancies, honoraria, stock ownership or options, expert testimony, royalties, or patents filed, received, or pending), are disclosed in an attachment.

☑ c. I certify that all financial and material support (including those providing data or access to patients, interviewees, etc.) for this research and work are clearly identified in an acknowledgement/disclosure section to be published with the manuscript.

☑ d. I certify that I agree with the description in the Acknowledgements/Disclosures statement in Section 5 of any support for the project or conflicts of interest as they pertain to myself.

**3. Other Disclosures.** Aside from financial interests, there may be other real or potential conflicts that need to be disclosed. In particular, we are concerned about situations in which an external sponsor, provider of data, or other entity may be able to influence either an author's ability to publish or to shape what he or she seeks to publish. We are also concerned that an author, or organizations with which he or she is affiliated, may have taken certain positions relevant to the research that should be disclosed. We may require that such information be publicly acknowledged. For the purposes of this disclosure, the term "contractual right" includes not only formal contracts and memoranda of understanding with outside parties, but also the rights of your employer to review and or approve your publications. Please check the appropriate boxes below and provide the information requested:

a. Sponsors and/or supporters of this research, e.g., organizations providing data, or supervisors within my own organization have:
   - ☐ i. a contractual right to review and approve the manuscript before submission or publication.
   - ☐ ii. a contractual right to review and comment on the manuscript within  days, after which it can be submitted without constraint.
   - ☐ iii. no contractual rights to review the manuscript before submission, but there is a requirement that the sponsor/supporter be given a copy of the accepted manuscript prior to publication.
   - ☐ iv. no requirement for prior approval or notification, but I solicited feedback and/or plan to provide an advance copy as a courtesy.
   - ☑ v. the manuscript has not been reviewed or commented upon by the sponsor(s) and I have no plans to provide advance notification.
   - ☐ vi. this research did not have a sponsor as defined above.

If you checked any of the boxes i through iv, please indicate the organizations involved and the circumstances. (For example, some organizations require that manuscripts undergo internal peer review for comment, but an author may publish any findings he or she wishes.)

IN THE SUBMITTED FORM, THE CORRESPONDING AUTHOR SHOULD PROVIDE DETAILS FOR EACH AUTHOR [NAMED] WHO HAS CHECKED ANY BOX i THROUGH iv.

| Please describe: |
|---|
| |

A contractual right by a sponsor, supporter, or employer to review and approve (and hence censor) a manuscript makes it appear to be a "work for hire" rather than research. On occasion such work may warrant publication in HSR, e.g., because it illustrates novel methods, but we will be reluctant to accept a manuscript with findings or conclusions that might be influenced by the ability of the sponsor to withhold permission to publish.

b. I have taken public stands (e.g., in print, media, expert witness, legislative testimony or other venues, with or without compensation) that are identified with a particular advocacy position relevant to the manuscript.

IN THE SUBMITTED FORM, THE CORRESPONDING AUTHOR SHOULD PROVIDE DETAILS FOR EACH AUTHOR [NAMED] WHO HAS CHECKED BOX b.

| Please describe: |
|---|
| |

c. The organization with which I am currently (or was, at the time of the manuscript's preparation) affiliated (as a spokesperson, board member, or similar prominent position) is identified with a particular advocacy position relevant to the manuscript.

IN THE SUBMITTED FORM, THE CORRESPONDING AUTHOR SHOULD PROVIDE DETAILS FOR EACH AUTHOR [NAMED] WHO HAS CHECKED BOX c.

| Please describe: |
|---|
| |

d. I certify I agree with the description in the Acknowledgements/Disclosures statement in Section 5 of disclosures as they pertain to myself.

**4.A. Author matrix.** Please complete the authorship matrix affirming which roles each author has fulfilled. To be listed as an author, persons should have made contributions in both of the shaded areas; all others should be acknowledged as contributors. In the box in 4.B., we invite you to name other contributors. In addition, you may use the box in 4.B. if you wish to name other kinds of contributions by authors.

Section 4 A. and B. will be made available in the electronic version of accepted manuscripts.

*You may wish to copy the author's names from the box on page 2.

| **Manuscript Number:** 14-0680 | **Each author must check at least one blue box showing important contributions to:** | | | **& check contributions in at least one orange box:** | |
|---|---|---|---|---|---|
| **Name of Each Author*** | **Conception and design** | **Acquisition of data (arranging for or obtaining)** | **Analysis and interpretation of data** | **Drafting the manuscript** | **Critical revision for important intellectual content** |
| James Marton | ✔ | ✔ | ✔ | ✔ | ✔ |
| Aaron Yelowitz | ✔ | ✔ | ✔ | ✔ | ✔ |
| Meredith Shores | ✔ | ✔ | ✔ | ✔ | ✔ |
| Jeffrey C. Talbert | ✔ | ✔ | ✔ | ✔ | ✔ |
| | ☐ | ☐ | ☐ | ☐ | ☐ |
| | ☐ | ☐ | ☐ | ☐ | ☐ |
| | ☐ | ☐ | ☐ | ☐ | ☐ |
| | ☐ | ☐ | ☐ | ☐ | ☐ |
| | ☐ | ☐ | ☐ | ☐ | ☐ |
| | ☐ | ☐ | ☐ | ☐ | ☐ |
| | ☐ | ☐ | ☐ | ☐ | ☐ |
| | ☐ | ☐ | ☐ | ☐ | ☐ |
| | ☐ | ☐ | ☐ | ☐ | ☐ |
| | ☐ | ☐ | ☐ | ☐ | ☐ |

**Section 4.B. Other Contributions**

Some research projects involve very extensive teams with many people who do not meet the criteria for authorship but who have contributed importantly to the work. The box below is designed to acknowledge other types of contributions. As appropriate, use this box to name other people who have made important contributions to the work reported in this manuscript. Please also name people who have played a significant role in preparing the manuscript but who are not listed as authors. NOTE: If you wish to acknowledge any of these contributors in print, please include their names and roles in the joint acknowledgment section below.

Please name contributors to be acknowledged for:

Statistical analysis

Data collection (under supervision)

Interviewing (under supervision)

Programming and data management

Editing for presentation or style

Obtaining funding

Administrative, technical, or material support

Supervision of research staff

Other (specify)

**INSTRUCTIONS: THE CORRESPONDING AUTHOR MUST FILL OUT SECTION 5.**

**5. Joint Acknowledgment/Disclosure Statement.** Please enter a 1-3 sentence statement that acknowledges all forms of financial and material support for the project, the roles of key individuals who should be recognized; (this may or may not extend to everyone listed as contributors). Please also include summary statements about what you feel are the conflicts of interests and disclosures without which some readers may feel that important relevant information is being withheld. The editors will determine whether these disclosures are sufficient or excessive and may return a revised version. All authors will need to approve the final version. As appropriate, please add at the end of your statement: 'Disclosures: None' or 'No Other Disclosures'.

Section 5 will be made available in both the electronic and print version of accepted manuscripts.

Joint Acknowledgement/Disclosure Statement:

James Marton is an employee of Georgia State University, while Aaron Yelowitz, Meredith Shores, and Jeffery C. Talbert are all employees of the University of Kentucky. We received no external funding related to this paper. We would like to thank Tim Harris, the staff at the Kentucky Cabinet for Health and Family Services, and two anonymous referees for their valuable comments. Any errors are, of course, our own.

**The corresponding** author should obtain permission to name all individuals named in an Acknowledgment or the Contributorship Section because readers may infer their endorsement of data and conclusions.

**The corresponding author must check the box below to affirm his/her certification that:**

- all persons who have made substantial contributions to the work reported in this manuscript (e.g., data collection, analysis, or writing or editing assistance) but who do not fulfill the authorship criteria are named with their specific contributions in the Contributorship Box associated with the manuscript.
- all persons named in the Contributorship box have provided me with permission to be named.
- no other persons have made substantial contributions to this manuscript.
- all authors have approved the Joint Acknowledgement/Disclosure Statement intended for publication and the author matrix.

☑ I, the corresponding author, certify that the above statements are true.

(Adapted, with permission, from the Journal of the American Medical Association, 2006)
